# Ventriculoarterial decoupling in human septic shock

**DOI:** 10.1186/cc13842

**Published:** 2014-04-24

**Authors:** Fabio Guarracino, Baldassare Ferro, Andrea Morelli, Pietro Bertini, Rubia Baldassarri, Michael R Pinsky

**Affiliations:** 1 Department of Anesthesia and Critical Care Medicine, University Hospital of Pisa, Via Paradisa 2, Pisa 56124, Italy; 2 Department of Anesthesiology and Intensive Care, University of Rome, “La Sapienza”, Viale del Policlinico 155, Rome 00161, Italy; 3 Department of Critical Care Medicine, University of Pittsburgh, 606 Scaife Hall, 3550 Terrace St, Pittsburgh, PA 15213, USA

## Abstract

**Introduction:**

Septic shock is the most severe manifestation of sepsis. It is characterized as a hypotensive cardiovascular state associated with multiorgan dysfunction and metabolic disturbances. Management of septic shock is targeted at preserving adequate organ perfusion pressure without precipitating pulmonary edema or massive volume overload. Cardiac dysfunction often occurs in septic shock patients and can significantly affect outcomes. One physiologic approach to detect the interaction between the heart and the circulation when both are affected is to examine ventriculoarterial coupling, which is defined by the ratio of arterial elastance (Ea) to left ventricular end-systolic elastance (Ees). In this study, we analyzed ventriculoarterial coupling in a cohort of patients admitted to ICUs who presented with vs without septic shock.

**Methods:**

In this retrospective cross-sectional opportunity study, we measured routine hemodynamics using indwelling arterial and pulmonary arterial catheters and transthoracic echocardiograms in 25 septic patients (group S) and 25 non–septic shock patients (group C) upon ICU admission. Ees was measured by echocardiography using a single-beat (Ees_SB_) method. Ea was calculated as 0.9 systolic arterial pressure/stroke volume, and then the Ea/Ees_SB_ ratio was calculated (normal value <1.36).

**Results:**

In group S, 21 patients had an Ea/Ees_SB_ ratio >1.36 (uncoupled). The four patients with Ea/Ees_SB_ ratios ≤1.36 had higher Ees_SB_ values than patients with Ea/Ees_SB_ ratios >1.36 (*P* = 0.007), although Ea measurements were similar in both groups (*P* = 0.4). In group C, five patients had uncoupled Ea/Ees_SB_ ratios. No correlation was found between Ees_SB_ and left ventricular ejection fraction and between Ea/Ees_SB_ ratio and mixed venous oxygen saturation in septic shock patients.

**Conclusions:**

Upon admission to the ICU, patients in septic shock often display significant ventriculoarterial decoupling that is associated with impaired left ventricular performance. Because Ea/Ees decoupling alters cardiovascular efficiency and cardiac energetic requirements independently of Ea or Ees, we speculate that septic patients with ventriculoarterial uncoupling may benefit from therapy aimed at normalizing the Ea/Ees ratio.

## Introduction

Septic shock is characterized in the resuscitated patient as a hyperdynamic, hypotensive cardiovascular state associated with multiorgan dysfunction and metabolic disturbances consistent with tissue dysoxia [1]. Importantly, peripheral vasodilation (relative hypovolemia and low systemic vascular resistance) can mask coexistent cardiac dysfunction [2,3]. Cardiac dysfunction in septic shock includes left ventricular (LV) diastolic and systolic dysfunction secondary to primary myocardial injury or right ventricular dysfunction due to pulmonary hypertension [4]. The combined loss of peripheral vasomotor tone and cardiac contractile impairment may potentially result in ventriculoarterial decoupling, the consequences of which can be worsening cardiac energetics and performance.

**Figure 1:**
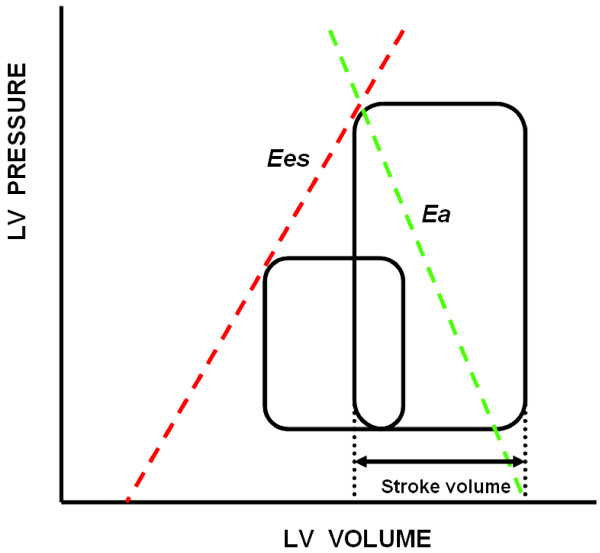
**Pressure–volume relationship in a cardiac cycle.** The slope of end-systolic elastance (Ees) (red line) represents the end-systolic pressure–volume relationship. The slope of arterial elastance (Ea) (green line) represents the relation between stroke volume (SV) and left ventricular (LV) systolic pressure as SV is varied.

Suga and Sagawa [5] and Sunagawa *et al*. [6] analyzed the circuit from the standpoint of LV ejection fraction (LVEF). In this model, LV contractility is described by the end-systolic pressure–volume relationship (ESPVR). The slope of the ESPVR, called end-systolic elastance (Ees), is a load-independent measure of cardiac contractility. End-systolic pressure is also a function of both LV stroke volume (SV) and a characteristic of the arterial outflow tract. The greater the SV for a given vascular tone, the greater the systolic arterial pressure. Similarly, increases in arterial systolic pressure for a constant preload and Ees will decrease SV and increase end-systolic volume. The slope representing the relationship between SV and systolic arterial pressure as SV is varied is called arterial elastance (Ea) (Figure 1). Thus, SV is both limited and defines end-systolic pressure through arterioventricular coupling [6]. Maximal myocardial efficiency, defined as the amount of external work performed for myocardial oxygen consumed, occurs when Ea is approximately one-half Ees [7] and has stronger dependence on Ea than on Ees [8]. Accordingly, the Ea/Ees ratio is a sensitive and independent estimate of cardiovascular efficiency [5]. A normally coupled human cardiovascular system has an Ea/Ees ratio = 1 ± 0.36 (median ± IQR), with normal values being 2.2 ± 0.8 mmHg/ml for Ea and 2.3 ± 1 mmHg/ml for Ees [9].

Uncoupling, defined as an Ea/Ees ratio >1.36, can result from changes in Ea, Ees or both. Uncoupling reflects a reduction in LV ejection efficiency, which can promote LV energetic failure. Although septic shock is associated with hypotension, which unloads LV ejection, it may, if unbalanced, result in Ea/Ees decoupling. We recently documented a primary decrease in peripheral impedance and an increase in peripheral compliance associated with a decrease in central aortic compliance in a porcine model of early endotoxic shock [10]. We hypothesized that similar Ea/Ees decoupling may also be present in septic patients and that this uncoupling may contribute to the observed impaired LV ejection effectiveness.

Ea is a lumped parameter of arterial tone which is influenced by arterial resistance and compliance, aortic impedance and systolic and diastolic time intervals. It reflects the net arterial load on LV ejection. Because end-systolic pressure follows maximal ejection pressure and end-systole occurs as LV relaxation starts, end-systolic pressure can be approximated as 90% of systolic arterial pressure and SV [5,6]. Similarly, Ees is defined as the slope of the LV ESPVR derived from a series of LV end-systolic pressure–volume points created by rapidly varying preload or afterload such that intrinsic contractility remains constant [6]. We previously showed that end-inspiratory hold maneuvers rapidly decrease preload, thus allowing the measurement of Ees [11]. Still, the need to rapidly alter either preload or afterload and also use invasive LV catheterization to measure LV volume has kept the bedside measurement of Ees out of the realm of routine bedside practice.

**Figure 2:**
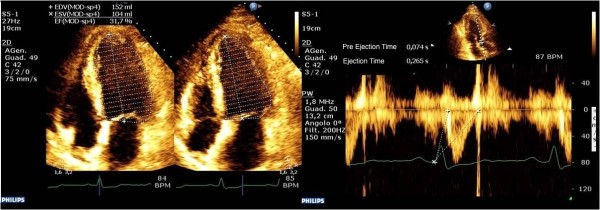
**Left ventricular end-systolic elastance was calculated by using the single-beat method.** These echocardiographic scans display the evaluation of ejection fraction (left image) and preejection and ejection time (right image) using aortic Doppler waveforms. Normalized ventricular elastance at arterial end-diastole (End) was measured according to the following formula: $\begin{array}{l} E_{\mathrm{es}\left(\mathrm{sb}\right)}=\left[P_{d}-\left(E_{\mathrm{Nd}\left(\mathrm{est}\right)}\times \;P_{s}\times \;0.9\right)\right]/[\mathrm{SV}\;\times \;E_{\mathrm{Nd}\left(\mathrm{est}\right)}]\\ E_{\mathrm{Nd}\left(\mathrm{avg}\right)}=\Sigma a_{i}\times \;t_{\mathrm{Nd}}\\ i=0\\ E_{\mathrm{Nd}\left(\mathrm{est}\right)}=0.0275–0.165\;\times \;\mathrm{EF}+0.3656\;\times \;\left(P_{d}/P_{\mathrm{es}}\right)+0.515\;\times \;E_{\mathrm{Nd}\left(\mathrm{avg}\right)} \end{array}$ where *a*_i_ values are 0.35695, -7.2266, 74.249, -307.39, 684.54. -856.92, 571.95 and -159.1 for *i* = 1 to *i* = 7, respectively. The value *t*_Nd_ value was determined by the ratio of pre-ejection period (R-wave to flow onset) to total systolic period (R-wave to end-flow), with the time of onset and termination of flow-defined Doppler. Systolic blood pressure and diastolic blood pressure were measured invasively. The single-beat method used to calculate left ventricular end-systolic elastance was previously validated by Chen *et al*. [8].

The demonstration that Ees can be estimated in a single beat (Ees_SB_) [12], and the subsequent validation of a noninvasive echocardiographic method to measure Ees_SB_[13] through the measure of LVEF, SV, pre-ejection time and systolic time interval when coupled with systolic and diastolic arterial pressure (Figure 2), made the clinical bedside measure possible. Thus, using these bedside estimates of Ea and Ees, we measured in patients presenting with vs without septic shock to analyze the presence of native ventriculoarterial decoupling.

## Methods

After we obtained approval from the ethical committees for human biomedical research at both the University Hospital of Pisa and the University of Pittsburgh, we conducted a retrospective cross-sectional study between July 2011 and February 2013 to elaborate hemodynamic data obtained from (1) patients presenting with septic shock as defined by the international consensus conference definition and treated them following the Surviving Sepsis Campaign guidelines [14] and (2) patients admitted to the ICU without presumed septic shock. Patients with a history of cardiac disease and preoperative cardiac surgery patients were excluded. Informed consent was obtained from all patients, and all were studied immediately after initial fluid resuscitation in accordance with the Surviving Sepsis Campaign guidelines, but prior to starting therapy with any vasoactive pharmacological agents.

We analyzed a series of measured hemodynamic variables and calculated parameters at the time of diagnosis of septic shock in our septic shock cohort (8 women and 17 men, age 69 ± 8 years (median ± IQR)) and at the time of ICU admission in our non–septic shock cohort with neurological problems but otherwise hemodynamically stable (11 women and 14 men, age 60 ± 11 years). These data included cardiac index (CI), heart rate and mean arterial pressure (MAP) invasively measured at a radial arterial site, as well as pulmonary artery occlusion pressure (P_pao_) and mixed venous oxygen saturation (SvO_2_) measured using a continuous cardiac output pulmonary artery catheter equipped with fiber optics (*Swan-Ganz oximetry thermodilution* catheter; Edwards Lifesciences, Irvine, CA, USA)_._

In nonseptic patients in whom a pulmonary artery catheter was not inserted at the discretion of the attending intensivist, the CI was measured using a pulse contour method (Vigileo Monitor/FloTrac Sensor System; Edwards Lifesciences), and central venous oxygen saturation (ScvO_2_) was measured instead of SvO_2_. None of the nonseptic patients were receiving vasoactive drug therapy at the time they were examined.

All patients in both groups had a transthoracic echocardiographic examination upon admission, all of which were performed by the same operator. Echocardiography was performed with a CX50 ultrasound system and an S5-1 Sector Array Transducer (Koninklijke Philips Electronics NV, Eindhoven, the Netherlands).

Parameters calculated using data gathered from the echocardiographic examination included LVEF, SV, preejection time and systolic time. Ees_SB_ was estimated by using the method of Chen *et al*. [13]. Ea was calculated as 0.9 × (systolic arterial pressure/SV), and the Ea/Ees_SB_ ratio was then calculated.

Statistical data are expressed as median ± IQR. Considering the small number of patients in our study, a nonparametric unpaired Mann–Whitney *U* test was used to compare groups. Spearman’s coefficient of rank correlation (ρ) was used to correlate variables. Fisher’s exact test was used in the analysis of contingency tables.

## Results

**Figure 3:**
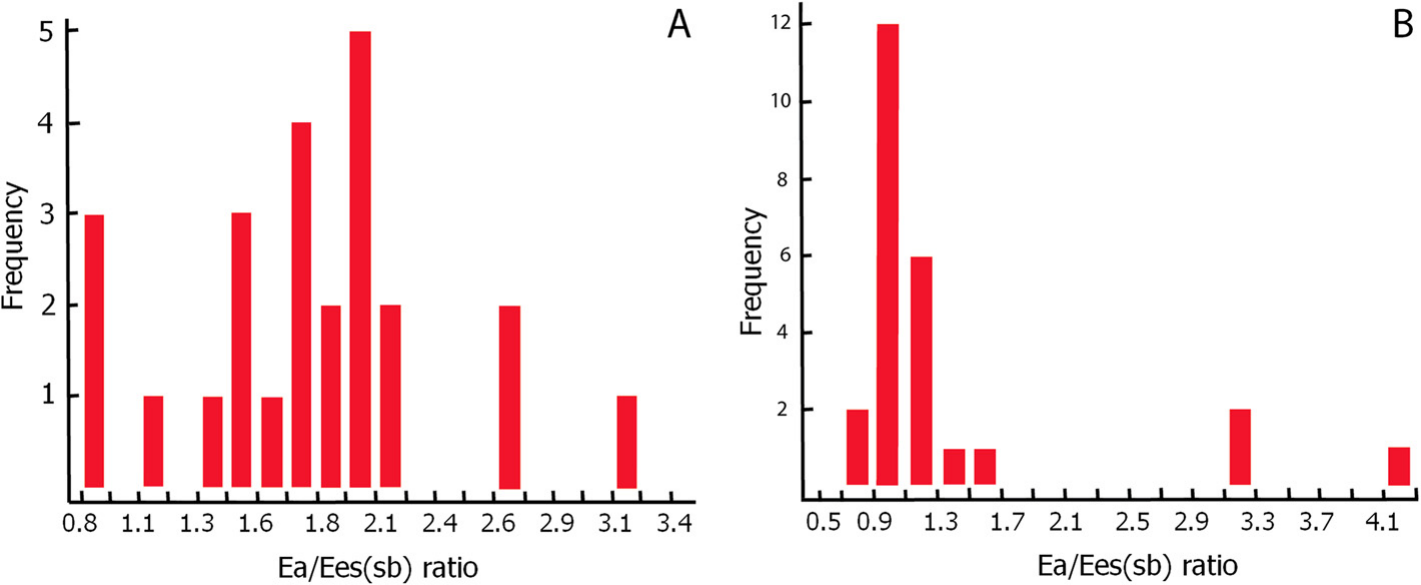
Graphs representing the distribution of ratios of arterial elastance to single-beat end-systolic elastance in newly diagnosed septic shock patients (A) and non–septic shock patients (B).

The data derived from 50 patients were analyzed. The 25 patients with a diagnosis of septic shock presented in a hyperdynamic (median CI = 2.77 L/min/m^2^ (IQR = 2.4 to 3.8) and HR = 115 beats/min (IQR = 109 to 124), hypotensive (MAP 58 IR 53–60) mmHg) state with a median P_pao_ of 13 mmHg (IQR = 10 to 14.2), median LVEF of 40% (IQR = 32.25 to 51.5), SvO_2_ of 60% (IQR = 58 to 69) and an Ea/Ees_SB_ ratio of 1.81 (IQR = 1.49 to 2.02) following initial fluid resuscitation. Only four patients had an Ea/Ees_SB_ ratio ≤1.36 at the time of diagnosis of septic shock, whereas the remaining twenty-one patients had an Ea/Ees_SB_ ratio >1.36 (Figure 3). The four patients with Ea/Ees_SB_ ratios ≤1.36 had higher Ees_SB_ compared with twenty-one patients with Ea/Ees_SB_ ratios >1.36 (*P* = 0.007), though Ea was similar in both the coupled and uncoupled septic shock patients (*P* = 0.4). We found no correlation between Ees_SB_ and LVEF (ρ = -0.0809, *P* = 0.7007) or between Ea/Ees_SB_ ratio and SvO_2_ in septic shock patients (ρ = -0.293, *P* = 0.15).

The 25 patients admitted in a non–septic shock state presented with a normal systemic flow state (CI = 2.8 L/min/m^2^ (IQR = 2.6 to 3)); HR = 80 beats/min (IQR = 71 to 104; MAP = 70 mmHg (IQR = 67 to 71) with an LVEF of 54% (IQR = 47.5 to 58), ScvO_2_ = 62% (IQR = 56.8 to 69) and Ea/Ees_SB_ ratio = 1.07 (IQR = 0.95 to 1.14). Five of these patients had a calculated Ea/Ees_SB_ ratio >1.36, and the remaining twenty patients had Ea/Ees_SB_ ratios <1.36 (Figure 3). Table 1 shows comparisons of variables between septic and nonseptic patients. Compared with non–septic shock patients, septic shock patients had reduced Ea; lower Ees_SB_, LVEF and MAP; and higher Ea/Ees_SB_ ratios and HRs. Table 2 shows the absence of correlation between Ea/Ees_SB_ status and normal or reduced LVEF in septic shock patients (*P* = 0.5) in a 2 × 2 contingency table.

**Table 1:** **Comparison of hemodynamic variables between septic shock and non–septic shock patients**^**a**^

**Measurements**	**Septic shock patients**	**Non–septic shock patients**	***P*-value**
**CI, L/min/m**^**2**^	2.7 (2.4 to 3.8)	2.8 (2.6 to 3)	0.76
**HR, beats/min**	115 (109 to 124)	80 (71 to 104)	<0.0001
**SAP, mmHg**	85 (75 to 92)	120 (95 to 135)	<0,0001
**MAP, mmHg**	58 (53 to 60)	70 (67.8 to 71.3)	<0.0001
**LVEF, %**	40 (32 to 52)	54 (48 to 58)	0.0098
**Ees**_**SB**_**, mmHg/ml**	0.7 (0.59 to 1.1)	2.1 (1.57 to 2.3)	<0.0001
**Ea, mmHg/ml**	1.4 (1.1 to 1.48)	2.3 (2.02 to 2.45)	<0.0001
**Ea/Ees**_**SB **_**ratio**	1.81 (1.49 to 2.03)	1.07 (0.95 to 1.18)	0.01

^a^*CI,* Cardiac index; *Ea,* Arterial elastance; *Ees*_*SB*_*,* Single-beat ventricular end-systolic elastance; *HR,* Heart rate; *LVEF,* Left ventricular ejection fraction; *MAP,* Mean arterial pressure; *SAP,* Systolic arterial pressure. Data are expressed as median and IQR. *P* < 0.05 was considered statistically significant.

**Table 2:** **Analysis of correlation between ventriculoarterial coupling and left ventricular ejection fraction in septic shock patients**^**a**^

	**LVEF normal (≥50%)**	**LVEF reduced (<50%)**
**Ea/Ees**_**SB **_**normal (<1.36),***n*	2	2
**Ea /Ees**_**SB **_**altered (>1.36),***n*	6	15

^a^*Ea,* Arterial elastance; *Ea/Ees*_*SB*_, Single-beat ventriculoarterial coupling; *Ees,* End-systolic elastance; *LVEF,* Left ventricular ejection fraction. *P* = 0.5 by Fisher’s exact test.

## Discussion

Our data show that, using bedside monitoring techniques [15], most of our patients who presented to ICUs in septic shock had significant ventriculoarterial decoupling independent of the commonly seen reduced Ea, whereas our non–septic shock patients displayed preserved ventriculoarterial coupling. This altered ventriculoarterial coupling in septic patients occurred despite a preserved or even elevated CI. These data suggest that, in septic shock patients, decoupling was associated with the observed impaired LV performance and reduced Ea. The lack of correlation between LVEF and Ees_SB_ suggests that LVEF cannot be considered a clinical index of contractility or ventricular performance. This conclusion is not surprising, because LVEF is a function not only of contractility but also of Ea. On the basis of these data, we speculate that the use of vasoconstrictors in septic shock with the aim of maintaining sufficient MAP according to the published Surviving Sepsis Campaign guidelines could be the cause of the reduced LVEF, as previously suggested [3].

Ventriculoarterial decoupling is an important index of cardiovascular inefficiency. An adequate Ea/Ees ratio is fundamental for efficient cardiovascular performance and is a determinant of cardiac energetics [16]. Ea/Ees ratio reflects the interaction between cardiac function and the arterial system that is necessary to modulate the cardiovascular response to either physiological or pathological conditions. The changes in CO and vascular resistance in different physiological conditions (for example, age, exercise, rest) and pathological conditions (for example, hypertension, heart failure, diabetes) strictly depend on both LV function and the arterial system. Aging and cardiovascular diseases such as hypertension, coronary artery disease, congestive heart failure and cardiac valve dysfunction can alter Ea/Ees coupling by reducing LV performance, increasing Ea or both [17].

Many researchers have shown that vasoactive drugs can affect ventriculoarterial coupling in several different clinical situations. The phosphodiesterase inhibitor E-1020 was found to increase heart mechanical efficiency by improving Ea/Ees coupling in heart disease patients [18]. Levosimendan, an inodilator, restored Ea/Ees coupling in patients with ischemic cardiomyopathy undergoing cardiac surgery [19], and enoximone improved Ea/Ees better than dobutamine in subjects with dilated cardiomyopathy [20,21]. Recently, Martin *et al*. demonstrated a better prognosis in patients with polytrauma using LV stroke work and ventriculoarterial coupling as targets of therapy [22].

Our study has some limitations. First, it is a retrospective analysis of prospectively collected data. Still, the data were collected in a consistent fashion from all participants. Second, the method we applied to measure Ees does not take into consideration the curvilinear shape of the elastance curve, which could be misleading if contractility was much reduced. However, estimated Ees values for our septic cohort were not depressed enough to reach this region of curvilinearity. Third, the definition of Ea was simplified as Ea = end-systolic pressure/SV, but this is routinely used as a valid surrogate. Furthermore, we measured arterial pressure from indwelling radial artery catheters that may either over- or underrepresent central aortic systolic pressure in septic patients. Differences of about 10 mmHg may exist between peripheral and central pressure sites. However, the mathematical effect of systolic arterial pressure on the calculated Ea/Ees_SB_ would be small because it is used in the calculation of both parameters. Fourth, we did not reanalyze our septic patients following restoration of arterial pressure with vasopressor agents or after recovery to see if the associated uncoupling was resolved. This latter limitation forms the basis of an ongoing clinical study.

We cannot deduce the clinical implications of this decoupling from our analysis; however, because most of our septic shock patients had uncoupled Ea/Ees at the time of diagnosis, we speculate that patients with septic shock and decoupled Ea/Ees would benefit from vasoactive therapies aimed at normalizing the Ea/Ees ratio. For example, in a murine model of sepsis, Ducrocq *et al*. [23] demonstrated that increasing MAP by the use of selective α-adrenergic vasopressors such as phenylephrine will unmask LV failure. Use of a more balanced α- and β-adrenergic agent, such as norepinephrine, may result in a better cardiovascular state with less potential for cardiac decompensation. Furthermore, the Surviving Sepsis Campaign guidelines suggest using inotropes as the last step in enhancing SvO_2_ values >70% [14]. We did not find a correlation between SvO_2_ values and ventriculoarterial coupling in our septic shock patients, which is not surprising, because, owing to possible alterations in peripheral oxygen uptake, high venous oximetry cannot be considered a good index of perfusion or cardiovascular performance in these patients.

## Conclusion

In septic shock patients, there is a higher percentage of ventriculoarterial decoupling compared to nonseptic patients admitted to ICUs. This decoupling is associated with impaired LV performance. Because ventriculoarterial decoupling is an index of cardiovascular inefficiency and a determinant of cardiac energetics, we speculate that such “uncoupled” patients may benefit from therapies aimed at normalizing the Ea/Ees ratio. However, this hypothesis remains to be tested.

## Key messages

• Ea and LV Ees can be measured at the bedside in critically ill patients.

• Septic shock affects both ventricular and arterial elastance.

• Disproportionate changes in either ventricular or arterial elastance will lead to ventriculoarterial decoupling.

• Therapies aimed at normalizing the Ea/Ees ratio may improve cardiovascular efficiency.

## Abbreviations

CI: Cardiac index; CO: Cardiac output; Ea: Arterial elastance; EesSBES: Single-beat end-systolic left ventricular elastance; Ees: End-systolic left ventricular elastance; EF: Ejection fraction; ESP: End-systolic pressure; ESPVR: End systolic pressure–volume relationship; HR: Heart rate; LV: Left ventricular; MAP: Mean arterial pressure; Ppao: Pulmonary artery occlusion pressure; ScVO2: Central venous oxygen saturation; SV: Stroke volume; SvO2: Mixed venous oxygen saturation.

## Competing interests

The authors declare that they have no competing interests.

## Authors’ contributions

FG made substantial contributions to the conception and design of the work, interpretation of data and drafting the manuscript and revising it critically for important intellectual content. BF Acquired, analyzed and interpreted the data and drafted the manuscript. AM made substantial contributions to the design of the work and revised the manuscript critically for important intellectual content. PB made substantial contributions to the conception or design of the work; acquired, analyzed and interpreted the data; and drafted the manuscript. RB made substantial contributions to the conception and design of the work and drafted the manuscript. MP Interpreted the data and revised the manuscript critically for important intellectual content. All authors read and approved the final manuscript.

## References

[B1] Werdan K Müller-Werdan U Elucidating molecular mechanisms of septic cardiomyopathy—the cardiomyocyte model Mol Cell Biochem 1996 163–164 291 303 10.1007/BF00408670 8974069

[B2] Flierl MA Rittirsch D Huber-Lang MS Sarma JV Ward PA Molecular events in the cardiomyopathy of sepsis Mol Med 2008 14 327 336 18256728 10.2119/2007-00130.Flierl PMC2227904

[B3] Vieillard-Baron A Septic cardiomyopathy Ann Intensive Care 2011 1 6 10.1186/2110-5820-1-6 21906334 PMC3159902

[B4] Parker MM McCarthy KE Ognibene FP Parrillo JE Right ventricular dysfunction and dilatation, similar to left ventricular changes, characterize the cardiac depression of septic shock in humans Chest 1990 97 126 131 10.1378/chest.97.1.126 2295231

[B5] Sunagawa K Maughan WL Sagawa K Optimal arterial resistance for the maximal stroke work studied in isolated canine left ventricle Circ Res 1985 56 586 595 10.1161/01.RES.56.4.586 3978773

[B6] Sunagawa K Maughan WL Burkhoff D Sagawa K Left ventricular interaction with arterial load studied in isolated canine ventricle Am J Physiol 1983 245 H773 H780 6638199 10.1152/ajpheart.1983.245.5.H773

[B7] Burkhoff D Sagawa K Ventricular efficiency predicted by an analytical model Am J Physiol 1986 250 R1021 R1027 3717375 10.1152/ajpregu.1986.250.6.R1021

[B8] Starling MR Left ventricular-arterial coupling relations in the normal human heart Am Heart J 1993 125 1659 1666 10.1016/0002-8703(93)90756-Y 8498308

[B9] Chantler PD Lakatta EG Najjar SS Arterial-ventricular coupling: mechanistic insights into cardiovascular performance at rest and during exercise J Appl Physiol 2008 105 1342 1351 A published erratum appears in *J Appl Physiol* 2009, **106:**1027 10.1152/japplphysiol.90600.2008 18617626 PMC2576043

[B10] Hatib F Jansen JRC Pinsky MR Peripheral vascular decoupling in porcine endotoxic shock J Appl Physiol 2011 111 853 860 10.1152/japplphysiol.00066.2011 21700890 PMC3174791

[B11] Kim HK Alhammouri MT Mokhtar YM Pinsky MR Estimating left ventricular contractility using inspiratory-hold maneuvers Intensive Care Med 2007 33 181 189 10.1007/s00134-006-0443-8 17103142

[B12] Takeuchi M Igarashi Y Tomimoto S Odake M Hayashi T Tsukamoto T Hata K Takaoka H Fukuzaki H Single-beat estimation of the slope of the end-systolic pressure-volume relation in the human left ventricle Circulation 1991 83 202 212 10.1161/01.CIR.83.1.202 1898642

[B13] Chen CH Fetics B Nevo E Rochitte CE Chiou KR Ding PA Kawaguchi M Kass DA Noninvasive single-beat determination of left ventricular end-systolic elastance in humans J Am Coll Cardiol 2001 38 2028 2034 10.1016/S0735-1097(01)01651-5 11738311

[B14] Dellinger RP Levy MM Rhodes A Annane D Gerlach H Opal SM Sevransky JE Sprung CL Douglas IS Jaeschke R Osborn TM Nunnally ME Townsend SR Reinhart K Kleinpell RM Angus DC Deutschman CS Machado FR Rubenfeld GD Webb SA Beale RJ Vincent JL Moreno R Surviving Sepsis Campaign Guidelines Committee including the Pediatric Subgroup Surviving Sepsis Campaign: international guidelines for management of severe sepsis and septic shock: 2012 Crit Care Med 2013 41 580 637 10.1097/CCM.0b013e31827e83af 23353941

[B15] Guarracino F Baldassarri R Pinsky MR Ventriculo-arterial decoupling in acutely altered hemodynamic states Crit Care 2013 17 213 10.1186/cc12522 23510336 PMC3672525

[B16] Prabhu SD Altered left ventricular-arterial coupling precedes pump dysfunction in early heart failure Heart Vessels 2007 22 170 177 10.1007/s00380-006-0954-9 17533521

[B17] Goto Y Futaki S Kawaguchi O Hata K Takasago T Saeki A Nishioka T Suga H Left ventricular contractility and energetic cost in disease models—an approach from the pressure–volume diagram Jpn Circ J 1992 56 716 721 10.1253/jcj.56.716 1495163

[B18] Takaoka H Takeuchi M Odake M Hayashi Y Mori M Hata K Yokoyama M Comparison of the effects on arterial-ventricular coupling between phosphodiesterase inhibitor and dobutamine in the diseased human heart J Am Coll Cardiol 1993 22 598 606 10.1016/0735-1097(93)90071-8 8335835

[B19] Guarracino F Cariello C Danella A Doroni L Lapolla F Stefani M Baldassarri R Vullo C Effect of levosimendan on ventriculo-arterial coupling in patients with ischemic cardiomyopathy Acta Anaesthesiol Scand 2007 51 1217 1224 10.1111/j.1399-6576.2007.01428.x 17850562

[B20] Ishihara H Yokota M Sobue T Saito H Relation between ventriculoarterial coupling and myocardial energetics in patients with idiopathic dilated cardiomyopathy J Am Coll Cardiol 1994 23 406 416 10.1016/0735-1097(94)90428-6 8294695

[B21] Beanlands RS Bach DS Raylman R Armstrong WF Wilson V Montieth M Moore CK Bates E Schwaiger M Acute effects of dobutamine on myocardial oxygen consumption and cardiac efficiency measured using carbon-11 acetate kinetics in patients with dilated cardiomyopathy J Am Coll Cardiol 1993 22 1389 1398 10.1016/0735-1097(93)90548-F 8227796

[B22] Martin RS Norris PR Kilgo PD Miller PR Hoth JJ Meredith JW Chang MC Morris JA Jr Validation of stroke work and ventricular arterial coupling as markers of cardiovascular performance during resuscitation J Trauma 2006 60 930 935 10.1097/01.ta.0000217943.72465.52 16688052

[B23] Ducrocq N Kimmoun A Furmaniuk A Hekalo Z Maskali F Poussier S Marie PY Levy B Comparison of equipressor doses of norepinephrine, epinephrine, and phenylephrine on septic myocardial dysfunction Anesthesiology 2012 116 1083 1091 10.1097/ALN.0b013e31824f9669 22407285

